# Risk Factors for Avascular Necrosis of the Femoral Head in Pediatric Femoral Neck Fractures

**DOI:** 10.7759/cureus.16776

**Published:** 2021-07-31

**Authors:** Sunny Chaudhary, Varun Garg, Dipun Mishra, Ramapriya Yasam, Sitanshu Barik, Sourabh K Sinha, Vivek Singh

**Affiliations:** 1 Orthopaedics, All India Institute of Medical Sciences, Rishikesh, IND

**Keywords:** open reduction, internal fixation, ratliff, delbet classification, screw, k-wire

## Abstract

Introduction

A neck of femur fracture is a rare injury in the pediatric population and is of foremost importance, as it is associated with a high rate of complications. It usually occurs due to high-velocity trauma or a fall from a height. There is a scarcity of data on risk factors and their role in the prognostication of avascular necrosis. The purpose of the study was to retrospectively analyze the association of various risk factors with avascular necrosis (AVN) of the femoral head in patients with a neck of femur fracture in the pediatric age group.

Material and methods

The study included 21 (13 males and 8 females) pediatric patients with a neck of femur fracture treated at a university-level hospital. The patients were followed for a minimum of one year and the clinico-radiological outcome was analyzed using Ratliff criteria. The association of AVN with age, gender, side, fracture type, and injury with treatment delay, type of reduction, and type of internal fixation used was studied.

Results

The mean age of the treated patients was 11 (±3.178) years (range=5-16 years). Avascular necrosis was seen in four patients and coxa vara occurred in two of them. A statistically significant association was seen between the Delbet fracture type and avascular necrosis, and three out of four cases of AVN were a Type I fracture (p-value=0.006). Three out of six patients having concomitant skeletal or other organ injuries developed AVN (p-value=0.022). The rate of AVN was higher in patients who were managed after 48 hours of initial injury but no statistically significant correlation was found (p-value=0.314). No statistically significant association with AVN was found between gender, age, type of reduction (closed/open), or the implant used (cannulated screws/k-wires).

Conclusions

Multiple independent factors may have a role in the development of AVN of the femoral head in children. Prognostication should not be based on a single factor. Statistically significant results in this study have shown that the type of fracture and concomitant skeletal or other organ injuries are important risk factors and should be kept in mind. All independent risk factors must be noted and should be considered while prognosticating the outcome of a child with a neck of femur fracture.

## Introduction

Femur neck fractures in children represent less than 1% of all pediatric fractures and are often due to a high-velocity injury [1-4]. There is a high rate of complications and associated skeletal injury and /or other organ system injury [5-6].

Vascularity of femoral head

Pediatric neck femur fractures are associated with a high rate of complications due to the presence of terminal blood supply to the capital femoral epiphysis. The three vessels supplying the femoral head are the medial femoral circumflex artery (MFCA), lateral femoral circumflex artery (LFCA), and artery of ligamentum teres (ALT). Trueta described that vascular supply to the femoral head is dynamic and changes with the age of the child [7]. Vascular supply has been divided into three stages based on the changes in capital femoral epiphysis [8]. The first stage starts from birth and ends with the appearance of the sub-capital epiphysis and is the tri-vessel stage in which the superior retinacular artery, inferior retinacular artery, and anterior retinacular artery supplies the femoral head. The second stage starts with the appearance of the physis and ends with its ossification. In this stage, the anterior retinacular artery does not cross the growth plate and only the inferior retinacular artery and superior retinacular artery are the supply to the capital femoral epiphysis. The third stage starts after the ossification of the physis [8].

Avascular necrosis (AVN) of the femoral head

AVN of the femoral head is one of the most dreadful complications and its rate varies from 0 to 77% in the literature [5,9]. Various independent risk factors having a role in the development of osteonecrosis have been studied such as the age of the patient, fracture type, displacement, time of injury to treatment, decompression of capsule, and reduction method [10]. There is still no consensus on the role of each factor in the development of osteonecrosis [11-13].

Various other complications seen in these fractures are malunion, delayed union, premature physeal closure, growth arrest, coxa vara, coxa valga, and infection. Definitive early treatment with anatomical reduction is imperative to achieve satisfactory outcomes while preventing complications [14-15].

Literature reports multiple risk factors for the development of AVN but with no consensus and no established causal relationships [10]. This study was conducted with the aim to describe the clinic-radiological outcomes in pediatric neck femur fractures in children and to evaluate various independent risk factors in the development of AVN.

## Materials and methods

A retrospective analysis of all children with a neck femur fracture from January 2017 to March 2019 was done after taking approval from the institutional ethical review board at a university-level teaching hospital. The inclusion criteria were children less than or equal to 16 years of age with a neck of femur fracture. Children with pathological fractures, open fractures, or nonunions were excluded from the study. Children with a follow-up of less than one year after surgery were also excluded.

**Figure 1:**
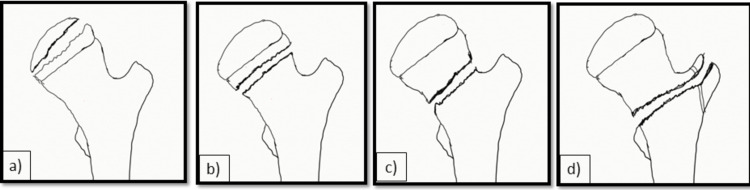
The Delbet Classification of Pediatric Proximal Femur Fractures a) Type 1 transepiphyseal fracture; b) Type II transcervical fracture; c) type III basicervical fracture; d) type IV intertrochanteric fracture

Medical records of all these patients were analyzed for demographic characteristics, mechanism of injury, associated injuries to other organ systems, fracture type, injury, and treatment duration. The type of reduction and method of fixation were also analyzed. The fracture type was classified according to Delbet’s classification (Figure 1) [16].

Treatment strategy

**Figure 2:**
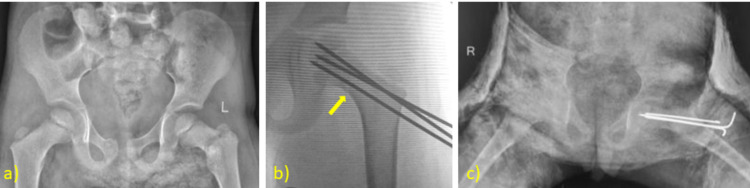
Case Example: Closed Reduction and K-Wire Fixation a) A five-year-old patient with Delbet type II fracture presented 115 hours after the injury and was taken to the operating room for fixation. Closed reduction was possible and stable fixation was achieved. b) The intraoperative c-arm image showing reduction and fixation. A yellow arrow is highlighting the fracture line. A hip spica cast was applied postoperatively. c) AP radiographs showing adequate reduction. AP: anteroposterior

**Figure 3:**
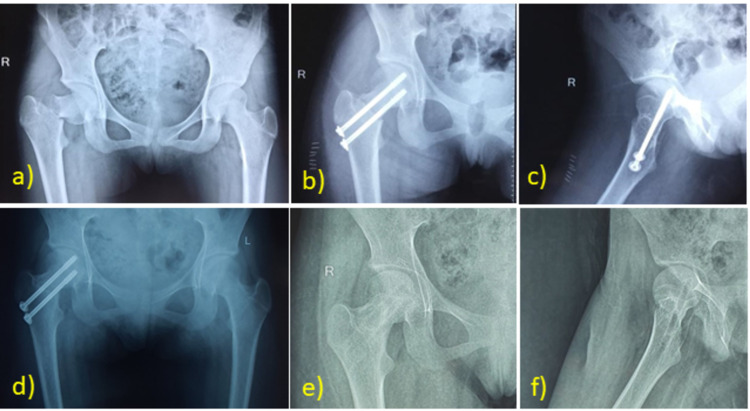
Case Example: Closed Reduction and Fixation With Screws A) AP radiograph of a 15-year-old female patient with Delbet type III fracture presented 14 hours after the injury and was taken immediately to the operating room for fixation. Postoperative AP (b) and lateral (c) radiographs of the proximal femur after closed reduction and internal fixation with cannulated screws through an anterolateral approach. d) AP radiograph showing union at three months follow-up. e) AP and (f) lateral radiographs demonstrating no signs of osteonecrosis after implant removal at one year. AP: anteroposterior

**Figure 4:**
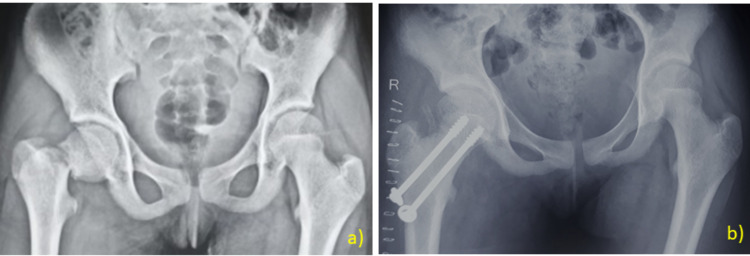
Case Example: Open Reduction and Fixation With Screws a) A 14-year-old female patient with Delbet type II fracture presented 12 hours after the injury. The patient was taken to the operating room and closed reduction was unsuccessful. Open reduction through the anterolateral approach and fixation with cannulated screws was done. b) Postoperative radiographs.

As per institutional protocol, emergent management of trauma patients was done. Definitive treatment was done after the stabilization of the patient. Closed reduction was attempted first, and open reduction was done after two attempts of failed closed reduction. Internal fixation was done using partially threaded cannulated cancellous screws. Kirschner wires were used for fixation in patients with age less than five years (Figures 2-4). A hip spica was applied for a minimum of six weeks in patients with age less than eight years. Postoperative radiographs of bilateral hip joints with pelvis were advised in all patients. The hip spica cast was removed at six weeks, and hip and knee range of motion exercises were started. Non-weight-bearing mobilization was started at 10 weeks and full weight-bearing was allowed once the union was seen on radiographs. The postoperative protocol was not rigid and was amenable to change based on fracture stability and clinical and radiological union achieved. Patients were followed every month till union was achieved, then every three months for a minimum period of one year. Implants were removed at six months to one year after complete consolidation at fracture site was seen.

Outcome assessment

The clinic-radiological outcome was assessed at the final follow-up using Ratliff's clinical and radiographic evaluation score (Table 1) [1].

**Table 1:** Ratliff Criteria for Functional Assessment of the Results of Treatment for Fracture of the Hip Source: [1]

Outcome	Clinical and Functional Evaluation
Good	Clinically, no or negligible pain, full or minimal restrictive hip movement, and normal activity or the avoidance of games. Normal or some deformity of the femoral neck in the radiograph.
Fair	Clinically, occasional pain, hip movement restriction less than 50%, and normal activity or the avoidance of games. Severe deformity of the femoral neck, mild avascular necrosis in the radiograph.
Poor	Clinically, disabling pain, hip movement restriction more than 50%, and restricted activity. Severe avascular necrosis, degenerative arthritis, arthrodesis in the radiograph.

**Figure 5:**
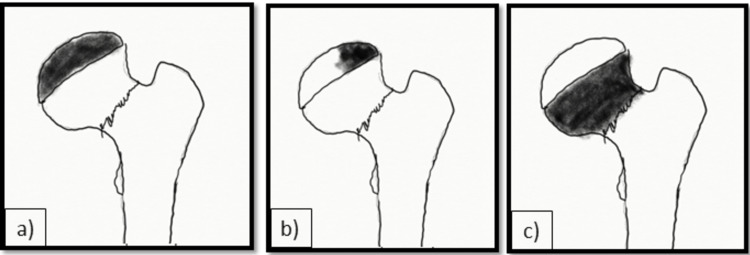
Ratliff's Classification of Avascular Necrosis a) Type I –Diffuse necrosis of femoral head and neck distal to the fracture. b) Type II- Necrosis confined to the epiphysis. c) Type III-Necrosis of the femoral neck with sparing of the epiphysis.

Radiographs were evaluated for fracture union, arthritic changes, neck-shaft angle, and avascular necrosis. The AVN was further classified according to the Ratliff classification system (Figure 5) [1].

Statistical analysis

Association of AVN with age, gender, side, fracture type, injury to treatment delay, type of reduction, and type of internal fixation used was studied. Evaluation of the risk factors was done using logistic regression analysis. A receptor operating characteristic curve (ROC) was used to determine the cut-off value in cases of a numerical variable. In categorical variables, subgroup analysis was done using logistic regression. Student’s t-test and chi-square tests were also used in subgroup analysis. A p-value of less than 0.05 was considered significant. All analyses were done using the Statistical Package for the Social Sciences software (SPSS software version 19; Armonk, NY).

## Results

Injury characteristics

Twenty-one patients (13 males and 8 females) with a neck of femur fracture and satisfying the inclusion criteria were studied. The patient's demographic profile and fracture characteristics were noted (Table 2). The mean age at injury was 11 (± 3.2) years (range 5-16 years). The mean follow-up was 13.76 (± 2.89) months (range 12-20 months). Six out of 21 (28.5%) patients had a concomitant injury to another organ system or extremity. Most of the patients had Delbet type II fractures (61.9%). Eighteen patients (85.7%) had a displaced fracture.

**Table 2:** Demographics and treatment characteristics

Variable	Cohort size (21)
Age (years) [range]	11 ± 3.178 (5-16)
Age (< or = 10 years) (years, %)	8 (38.1%)
Age (> 10 years) (years, %)	13 (61.9%)
Gender (number, %)	
Female	8 (38.1%)
Male	13 (61.9%)
Side of injury (number, %)	
Right	13 (61.9%)
Left	8 (38.1%)
Injury mechanism (number, %)	
Road traffic accident	11 (52.3%)
Fall from height	6 (28.5%)
Sports-related injury	4 (19.0%)
Associated Injury (number, %)	6 (28.5%)
Displaced fractures (number, %)	18 (85.71%)
Delbet type (number, %)	
I	4 (19%)
II	13 (61.9%)
III	4 (19%)
Time to reduction (mean delay in hours) (range)	88.76 (14-355)
Time to reduction (< or = 48 hours) (number, %)	10 (47.6%)
Time to reduction (>48 hours) (number, %)	11 (52.4%)
Type of reduction (number, %)	
Closed reduction	9 (42.9%)
Open reduction	12 (57.1%)
Type of internal fixation (number, %)	
Cannulated screws	20 (95.2%)
Kirschner wires	1 (4.8%)

Operative characteristics

The mean time from initial injury to surgery was 88.76 hours (range 14-355 hours). The most common reason long injury to surgery duration was delayed presentation. Few patients had in-hospital delays to ensure the safety of children with severe combined injury or unstable circulatory conditions. All patients underwent definitive treatment either by open reduction (12 patients) or close reduction (9 patients) with internal fixation using partially threaded cannulated cancellous screws (20 patients) or Kirschner wires (1 patient).

Operative outcome

All fractures united with two (9.52%) going into varus malunion. These patients did not report any pain, limitation of movement, or avoidance of any other activity.

**Figure 6:**
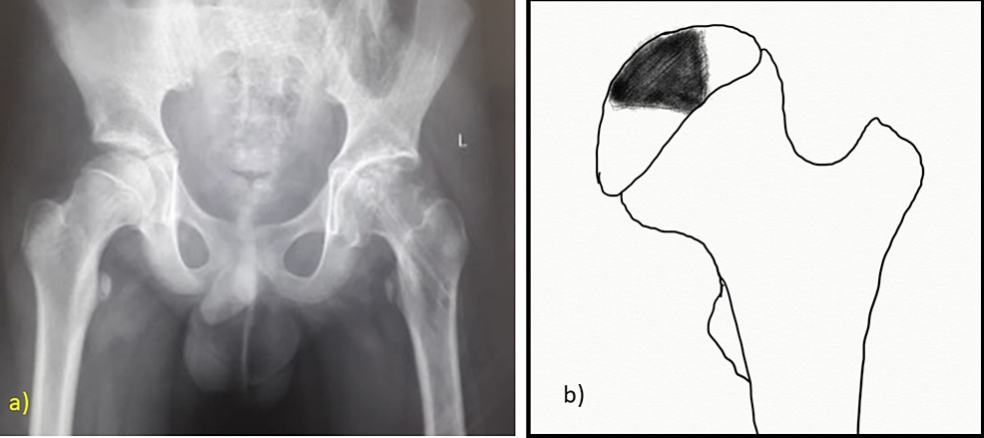
Case Example: Segmental Avascular Necrosis of Femoral Head A 12-year-old male: closed reduction and screw fixation were done. a) Radiographs one month after screw removal showing segmental involvement of the head (Ratliff type II). b) Pictorial representation of the areas of avascular necrosis of the femoral head.

**Figure 7:**
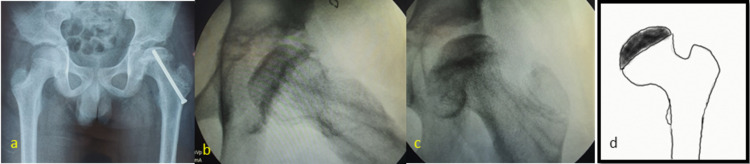
Case Example: Global Avascular Necrosis of the Femoral Head An 11-year-old male: closed reduction and screw fixation were done. a) Intraoperative C-arm images after screw removal showing global involvement of the head (Ratliff type I). b) Pictorial representation of complete involvement of the femoral head.

AVN was seen in four out of 21 cases (19.04%) and three of them had segmental involvement (Ratliff type II AVN) (Figure 6) while one had global involvement (Ratliff type I AVN) of the femoral head (Figure 7).

The hip outcome was good in 17 (80.9%) cases, three (14.28%) of them had a fair outcome, and one (4.76%) had a poor outcome based on Ratliff’s radiographic and functional evaluation score. All of the patients (4/21) with fair and poor outcomes had developed avascular necrosis. These patients had occasional pain with mild hip movement restriction and were able to carry out the normal activities.

It was also found that three out of four patients who developed AVN had sustained a Delbet type 1 fracture and chances of developing this complication were found to be significantly more in this fracture pattern than others (p-value=0.006) (Table 3).

**Table 3:** Patient and fracture characteristics with overall complications and avascular necrosis (AVN)

Characteristics	Complications (AVN +Coxa vara)			AVN		
	Present (n=6)	Absent (n=15)	p-value	Present (n=4)	Absent (n=17)	p-value
Gender (no, %)			0.776			0.081
Male	4 (30.8%)	9 (69.2%)		4 (30.8%)	9 (69.2%)	
Female	2 (25%)	6 (75%)		0 (0%)	8 (100%)	
Age (years) [range]			0.776			0.549
Age (< or = 10 years)	2 (25%)	6 (75%)		1 (12.5%)	7 (87.5%)	
Age (> 10 years)	4 (30.8%)	9 (69.2%)		3 (23.1%)	10 (76.9%)	
Associated Injury (no, %)			0.015			0.022
Present 6 (28.57%)	4 (66.7%)	2 (33.3%)		3 (50%)	3 (50%)	
Absent 15 (71.43%)	2 (13.3%)	13 (86.7%)		1 (6.7%)	14 (93.3%)	
Delbet type (no, %)			0.049			0.006
I	3 (75%)	1 (25%)		3 (75%)	1 (25%)	
II	3 (23.1%)	10 (76.9%)		1 (7.7%)	12 (92.3%)	
III	0 (0%)	4 (100%)		0 (0%)	4 (100%)	
Time to reduction (hours) (range)			0.89			0.314
Time to reduction (< or =48 hours)	3 (30%)	7 (70%)		1 (10%)	9 (90%)	
Time to reduction (>48 hours)	3 (27%)	8 (72.7%)		3 (27.3%)	8 (72.7%)	
Type of reduction (no, %)			0.13			0.422
Closed reduction (no, %)	1 (11.1%)	8 (88.9%)		1 (11.1%)	8 (88.9%)	
Open reduction (no, %)	5 (41.7%)	7 (58.3%)		3 (25%)	9 (75%)	
Type of internal fixation (no, %)			0.517			0.619
Cannulated screws (no, %)	6 (30%)	14 (70%)		4 (20%)	16 (80%)	
Kirschner wires (no, %)	0 (0%)	1 (100%)		0 (0%)	1 (100%)	

Three out of four patients who developed this complication had associated injuries to other organ systems or additional fractures (Table 3). On statistical analysis, it was found that cases with associated injuries had a higher chance of developing AVN than those without (p-value=0.022) (Table 3).

No statistically significant association with AVN was found between gender, age, side, time to reduction (injury to treatment delay in hours), type of reduction (closed/open), or the implant used (cannulated screws/k-wires) (Table 3). Various risk factors assessed in cases that developed AVN have been tabulated in Table 4.

**Table 4:** Associated injuries, their management and outcome AVN: avascular necrosis; RTA: road traffic accident

S. No	Age/Gender		Mechanism of Injury	Associated Injury	Treatment	Complications
1	5y/Female		RTA	Ipsilateral closed fracture: tibia and fibula	Closed reduction: spica	Coxa vara
2	6y/Male		Fall from height	Temporal bone fracture	Conservative	None
				Liver laceration (grade2)	Conservative	
3	9y/Male		RTA	Contralateral closed fracture shaft of the femur	Submuscular plating	AVN
				Splenic laceration (grade 5)	Splenectomy	
				Liver laceration (grade2)	Conservative	
4	11y/Male		RTA	Ipsilateral closed fracture: tibia and fibula	Closed reduction: spica	AVN
5	11y/Male		RTA	Contralateral open fracture shaft of femur (Gustilo Anderson type 1)	External fixator: TENS	None
				Ipsilateral inferior pubic rami fracture	Conservative	
6	12y/Male		RTA	Contralateral open fracture: tibia and fibula	External fixator	AVN

## Discussion

Femur neck fractures are infrequently seen in children and a great amount of force is required to produce them. Most commonly (in approximately 50%), the injury is sustained after high-velocity trauma [17]. Risk factors for the development of AVN are diverse. Studies have suggested patient’s age, gender, type of fracture, capsular decompression, time to surgery, and method of fixation as independent risk factors for the same [6,12-13,18-19].

Fracture type

In the present study, the Delbet type II fracture was the most common fracture pattern (47.61%). Dai et al. in their study of 44 children reported that more than half (25 out of 44) were of Delbet type II [6]. Another systematic review and meta-analysis by Khatib et al which included 231 pediatric femoral neck fractures also reported that type II was seen in more than 50% of the cases [19].

Avascular necrosis

AVN is the most critical factor that determines the prognosis of neck femur fractures. It is the most dreaded complication of this fracture and lacks effective treatment. The rate of AVN in this study was 19.04% (4/21). The rate of AVN of the femoral head reported in other studies varies from 0% to 77% [5,9].

Age and AVN

A study by Moon ES et al. has shown that AVN was more common in the adolescent age group than in young children (30% vs 19%) [10]. A similar study of 52 fractures by Pforringer et al. also shows increased chances of AVN with age [20]. Another study by Kay et al. reported that six of the nine cases of AVN were of age more than 10 years [21].

In this study, three out of four cases of AVN were of age greater than 10 years but no statistically significant results were seen (p-value=0.55).

Fracture type and AVN

In a meta-analysis of 360 patients by Moon ES et al., the occurrence of AVN was 38% of type I, 28% of type II, 18% of type III, and 5% of type IV fractures [10]. Another study by Canale et al. has reported incidences as high as 100% in type I, 90% in type II, 27% in type III, and 14% in type IV fractures [16].

Similar results were found in this study, and three out of four patients who developed AVN had sustained a Delbet type 1 fracture. The chances of developing this complication were found to be significantly more in this fracture pattern than others (p-value =0.006) (Tables 3-4).

Associated injuries and AVN

Neck of femur fractures are often associated with injury to other bones or other organ systems in the body. In this study, a concomitant injury was observed in six patients (28.57%). Dai et al. reported similar findings and combined injuries were found in 23% of the cases but there was no statistically significant result showing their association with the development of AVN [6]. Contrary to the previous study, in our study, three out of four patients who developed AVN had associated injuries and they were a risk factor for its development (p-value=0.022).

The vascular damage to the supply of the femoral head is considered to take place during initial injury and fracture fragment displacement and is supported by few studies [12,22]. Associated injuries give us an indirect way to know the severity of initial trauma and thus may help in prognostication. 

Injury to treatment duration (delay) and AVN

A study by Azam MQ et al. reported that there was a progressive increase in the incidence of AVN with delay in weeks following initial injury [23]. They reported that the rate of AVN was 22.22% of patients operated on in the first week, 46.15% in the second week, and 60% in those who were operated on in the third week after injury.

Similar findings were noted in this study, three out of four cases who developed AVN were operated on after 48 hours of injury. Even though the number of cases who developed AVN was more in late surgeries but the result was not statistically significant.

Yeranosian M et al. in their systematic review of 30 studies comprising 935 patients reported that the rate of AVN was 4.2 times higher in patients who were operated on after 24 hours of injury than in patients who were operated on early [24].

Early hip decompression and AVN

In a study by Bukva et al., early decompression of the hip has been shown to decrease the incidence of AVN in patients with fracture of the neck of the femur in the pediatric age group [25]. Patterson et al. reported that the literature fails to show the role of early hip decompression in reducing the occurrence of osteonecrosis [26].

Yeranosian M et al. in their systematic review of 935 patients also did not show a beneficial effect of capsular decompression on the rate of AVN [24].

Type of reduction and AVN

In this study, the percentage of AVN was 25% (3 out of 12) in those who underwent open reduction as compared to 11% (1 out of 9) in cases who were managed with closed reduction. The difference was not statistically significant (p-value=0.42). Recent studies have suggested a lower incidence of AVN in patients treated with open reduction and internal fixation as compared to conservative management [24,27-28]. The rationale behind open reduction is that it aids in the anatomic reduction and helps in capsular decompression. Few studies have advocated early decompression of the hip capsule to decrease the rates of osteonecrosis [27-28].

Many surgeons desist from open reduction to avoid injury to the blood vessels supplying the femur head but the lateral epiphyseal vessel, a terminal branch of the medial circumflex femoral artery, runs along the neck and not in the capsule and therefore is not injured while doing anterior capsulotomy during open reductions. Ju et al. in their study of 58 patients reported that open reduction and internal fixation had better outcomes than closed reduction and internal fixation [29].

Complications

Coxa vara was previously reported was found to be in 10% to 20% of children with a neck femur fracture [1,29]. It is the second most common complication after avascular necrosis of the femoral head [30]. The reason for coxa vara is the loss of reduction and progression of the deformity. In our study, coxa vara was found in two (9.52%) of the patients and was similar to studies reported in the literature.

Previous studies have shown a non-union rate of 7%-10% in children with a neck femur fracture with predisposing factors like oblique fractures, type II and III fracture, inadequate fixation, and infections. Non-union of femur neck fracture was not seen in any patient in our study.

According to previous studies, postoperative infection was found in 1% of patients, but it was not observed in any of the patients in our study. 

The limitations of the study are the small sample size and being a retrospective study with a short follow-up. Considering that only four patients developed AVN, the study is not adequately powered to predict the risk of AVN. Further large multicentered studies are needed to assess the association of various risk factors.

## Conclusions

Multiple independent factors may have a role in the development of avascular necrosis (AVN) of the femoral head in children. Prognostication should not be based on a single factor. Statistically significant results in this study have shown that the type of fracture and associated injuries are important risk factors and should be kept in mind. All independent risk factors must be noted and should be considered while prognosticating the outcome of a child with a fracture of the neck of the femur. Authors feel that to know the association of each independent risk factor better, there is a need for further, large sample-sized, multi-centric, prospective studies. Large sample size will help us decrease the margin of error and generalize results to the pediatric population.
